# Follicle Development of Xenotransplanted Sheep Ovarian Tissue into Male and Female Immunodeficient Rats

**DOI:** 10.22074/ijfs.2015.4551

**Published:** 2015-10-31

**Authors:** Leila Sadat Tahaei, Hussein Eimani, Ghazaleh Hajmusa, Rouhollah Fathi, Mojtaba Rezazadeh Valojerdi, Abdolhossein Shahverdi, Poopak Eftekhari-Yazdi

**Affiliations:** 1 Department of Embryology, Reproductive Biomedicine Research Center, Royan Institute for Reproductive Biomedicine, ACECR, Tehran, Iran; 2 Department of Anatomy, Faculty of Medicine, Baqiyatallah University, Tehran, Iran; 3 Animal Core Facility, Reproductive Biomedicine Research Center, Royan Institute for Biotechnology, ACECR, Tehran, Iran

**Keywords:** Follicle, Rat, Sheep, Xenotransplantation

## Abstract

**Background:**

This study aimed to assess follicle survival after xenotransplantation of sheep ovarian tissue into male and female immunodeficient rats. We evaluated the effects of gonadotropin treatment on follicular development in the transplanted tissue.

**Materials and Methods:**

In this experimental study, sheep ovarian cortical strips were transplanted into the neck back muscles of 8 male and 8 female immunodeficient, castrated rats. Fourteen days after surgery, each rat was treated with human menopausal gonadotropin (hMG) for 9 weeks. One day after the last injection, ovarian tissues were removed and fixed for histology assessment. Histology analyses were performed before and after grafting. Estradiol (E_2_) levels were measured before and after gonadectomy, and at the end of the experiment. The control group consisted of 7 male and 7 female noncastrated/non-grafted rats and the sham group comprised 7 male and 7 female castrated/ non-grafted rats for comparison of serum E_2_ concentrations.

**Results:**

The percentage of primordial follicles decreased after transplantation in male (25.97%) and female (24.14%) rats compared to the control group (ovarian tissue nongrafted; 37.51%). Preantral follicles increased in the male (19.5%) and female (19.49%) transplanted rats compared to the control group (11.4%). Differences in antral follicles between male (0.06 ± 0.0%) and female (0.06 ± 0.0%) rats were not noticeable compared to control (1.25 ± 0.0%) rats. We observed a significantly higher percent of mean E_2_ secretion in grafted males compared to grafted females (P˂0.05).

**Conclusion:**

Despite significant differences in E_2_ secretion between xenografted male and female rats, we observed no statistical differences in terms of follicular development.

## Introduction

Transplantation of a reproductive organ is one treatment for sterility. Transplantation of vitrifiedwarmed ovarian tissue from the donor female to herself can revive fertility. Inability to transplant ovarian tissue from the donor female or risks of disease relapse often prohibit autotransplantation (1,3). Xenotransplantation can remove transfer risk or relapse of cancer cells that may occur with autotransplantation. Xenotransplantation of ovarian tissue not only provides the possibility of access to gametes for reproduction from vitrified ovarian tissue, but also acts as a tool to understand the mechanism of follicular development (4,8). Van den Broecke et al. (9) have shown that follicular growth reduced 48 hours after xenotransplantation through hypoxia before angiogenesis. In order to resolve this problem, some researchers advocate the use of hormone therapy (2,9). Different gonadotropins at several doses are used for resumption of follicle growth in transplants. In mammals, development of antral follicles during the ovulatory phase is dependent on gonadotropin secretions of luteinizing hormone ( LH ) and follicle-stimulating hormone ( FSH ) by the anterior pituitary gland (9). However, other studies have reported the combined effect of gonadotropins exogenous injection in mice castrated have the same effect as those produced by the pituitary gland. Injections of gonadotropins have the same effect as those produced by the pituitary gland (2,10,13). Maltaris et al. (14) reported that increasing the dose of human menopausal gonadotropin ( hMG ) hormone injection two weeks after transplantation in gonadectomy mice resulted in better growth of primary follicles up to the antral follicle stage. 

The present study aimed to determine the optimal transplant conditions for maintenance, growth and maturation of follicles after xenotransplantation of fresh ovarian tissue from sheep into immunodeficient rats. We evaluated the ovarian tissue response to exogenous gonadotropin stimulation. 

## Materials and Methods

To follow-up the current experimental study, all chemicals were purchased from Sigma ( USA ) and Gibco ( Life Technologies Ltd., UK ), with the exception of hMG and human serum albumin ( HSA ) which were procured from Organon, Oss, Holland and from Biotest, Germany, respectively. All animal procedures were approved by the Ethical Committee at Royan Institute. 

### Sheep ovarian tissue preparation

Sheep ovaries were procured from a slaughterhouse in Shahriar ( Iran ). Ovaries were transported in phosphate buffered saline medium ( DPBS, 14190, Gibco ) supplemented with 50 mg/ml streptomycin and 60 IU/ml penicillin ( Gibco ) at 39˚C. In the laboratory, the ovaries were washed three times with fresh DPBS and placed on a sterile Petri dish that contained HEPES tissue culture medium ( HTCM ) supplemented with 10% HSA. Next, we separated the medulla from the cortex. The cortex was cut into slices with approximate dimensions of 1 mm ( length )×2 mm ( width )×2 mm ( thickness ). Sections were placed in fresh HTCM medium supplemented with 10% HSA. The ovarian pieces were washed three times with TCM. One or two pieces of ovarian cortex were fixed in Bouin’s solution as a fresh control for morphological and functional analyses (15). 

### Experimental design

We evaluated the host gender ( male vs. female rats ) on follicular development in xenografted sheep ovarian tissue. In this study, 8 sheep ovaries were used. Each ovary was segmented into 3 parts and divided into 3 groups, a control ( intact nongrafted ) and two experimental ( XM=xenografted in male and XF=xenografted in female ). The control group consisted of 7 male and 7 female noncastrated/non-grafted rats and the sham group comprised 7 male and 7 female castrated/nongrafted rats for comparison of serum estradiol (E_2_) concentrations. 

### Immunosuppressed rats

Sixteen female and male Wistar rats were housed in standard cages under a 12-hour light/ dark regime at 24-27˚C. The rats were immunesuppressed by the addition of 210 mg/l of cyclosporine-A ( Sandimmune, Novartis Pharmaceuticals, USA ) to their drinking water for five days before xenotransplantation of the ovarian tissues. The rats remained on the same immunosuppressant throughout the experiment. Rats had free access to sterilized food and water. Serum levels of cyclosporine with the average of concentration 1750 ± 34 ng/ml were measured in a random sample of rats (16). 

### Castrated rats

The immunosuppressed rats were anesthetized with intraperitoneal ( i.p. ) injections of 50 mg/kg bodyweight of 10% ketamine ( Alfasan, Woerden, Holland) and 5 mg/kg xylazine ( 2%, Alfasan, Woerden, Holland ). Gonadectomy was performed through a dorsomedian incision in female rats and through a ventromedial incision in male rats. 

### Transplantation

For transplantation, we used anesthetized, castrated rats. Surgery procedures were carried out under a laminar flow hood under aseptic conditions. One piece of ovarian tissue was placed in each rat’s neck muscle. The muscle was subsequently sutured with absorbable thread. 

### Hormone stimulation

Each female and male rat received i.p. injections of hMG ( 5 IU FSH / 5 IU LH ) every second day starting from day 14 after transplantation for 9 consecutive weeks. This dose was adjusted from an earlier study by Kagabu and Umezu (17). 

### Estradiol determination

We measured E_2_ levels before and after the gonadectomy, and at the end of the experiment. Rats were anesthetized, after which 800-1000 μl of blood was obtained using an orbital sinus puncture technique for E_2_ measurement. Blood was allowed to clot at room temperature (24˚C) for 30 minutes, and then centrifuged at 3000×g for 5 minutes for serum collection. E_2_ was determined by an ELISA Kit (ABIN416279) according to the manufacturer’s instructions.

### Histological assessment

Ovarian tissue from the experimental ( XM, XF ) and control ( intact non-grafted ) groups were fixed in Bouin’s solution, embedded in paraffin wax, serially sectioned at 6 μm, and stained with hematoxylin and eosin. The numbers of morphologically normal and degenerated follicles were counted in all prepared slides from each group, which included 24 total sections. All sections were studied using a light microscope ( Olympus CX31, Philippines ) at a magnification of ×200. According to Liu et al. (18) "follicles were classified as follows: i. Primordial follicles with one layer of flattened pregranulosa cells that surrounded the oocytes; ii. Primary follicles with one layer of cuboid granulosa cells, iii. Preantral follicles with two or more layers of granulosa cells and no antrum and iv. Antral follicles with an antral cavity (17)". Eosinophilia of the ooplasm, and wrinkling of nuclear membrane of the oocytes were considered as signs of atresia (15). 

### Statistical analyses

Percentages of growing follicles and E_2_ concentrations in each group were analyzed using oneway ANOVA. Numbers of follicles between experimental groups were analyzed according to the Kruskal-Wallis test. A value of P<0.05 was considered statistically significant. 

## Results

### Surgery recovery

Tissue from 16 sheep ovaries were transplanted into 16 gonadectomy rats ( 8 male and 8 female ). However, after surgery, 7 male and 6 female rats survived. Therefore we evaluated tissue from 7 control group ovaries ( ovarian tissue non-transplanted ), 6 ovaries transplanted into female rats, and 7 ovaries transplanted into male rats. 

### Histological evaluation

**Fig.1:**
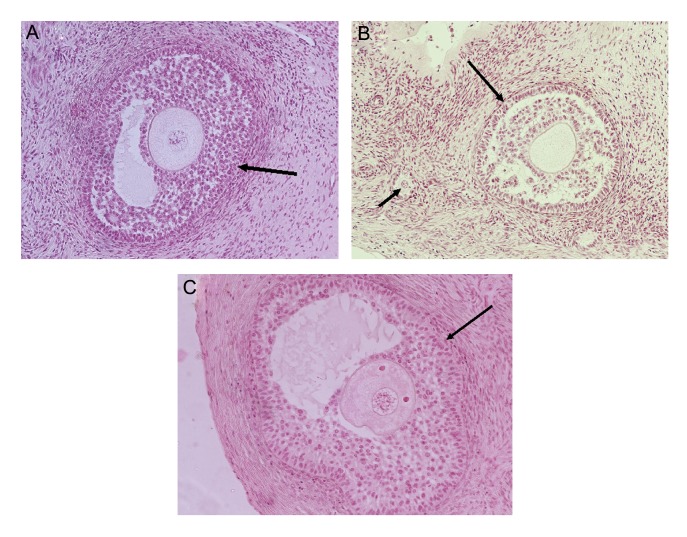
Hematoxylin–eosin staining. Histology staining of antral follicles in A. Control group (ovarian tissue non-grafted), B. Xenotransplanted sheep ovarian tissue into females (large arrows; Antral follicle and small arrow; Primary follicle) and C. Xenotransplanted sheep ovarian tissue into males (magnification: ×200).

Histological assessment of the ovarian tissues showed a non-significant decrease in the percentage of primordial follicles in the XM ( 25.97 ± 0.03% ) and XF ( 24.14 ± 0.03% ) groups compared to the control group ( 37.51 ± 0.05% ). The percentage of primary follicles non-significantly increased in both the XM ( 47.7 ± 0.03% ) and XF ( 48.85 ± 0.04% ) groups compared to the control group ( 43.22 ± 0.04% ). The lowest percentage of preantral follicles was in the control group ( 11.4 ± 0.03% ) compared to the XM ( 19.5 ± 0.02% ) and XF ( 19.49 ± 0.02% ) groups. There were no significant differences in preantral follicles between the groups. There was no significant difference in terms of follicle degeneration among the groups (Table 1, Fig.1). Differences of antral follicles in XM ( 0.06 ± 0.0% ) and XF groups ( 0.06 ± 0.0% ) were not noticeable compared to the control group ( 1.25 ± 0.0% ). 

**Table 1:** Mean percentage of viable follicles from sheep ovarian tissue grafts at nine weeks after xenotransplantation among the control, male xenograft (XM), and female xenograft (XF) groups


Follicles	Primordial (%)		Primary (%)		Preantral (%)		Antral (%)	
Experimental groups	Normal	Degenerated	Normal	Degenerated	Normal	Degenerated	Normal	Degenerate

**Control(non-grafted)**	37.51±0.05	2.07±0.0	43.22±0.04	3.28±0.0	11.4±0.03	0.85±0.0	1.25±0.0	0.0±0.0
**XM**	25.97±0.03	2.01±0.01	47.7±0.03	2.92±0.01	19.5±0.02	1.41±0.0	0.06±0.0	0.0±0.0
**XF**	24.14±0.03	1.66±0.0	48.85±0.04	4.0±0.0	19.49±0.02	1.31±0.0	0.06±0.0	0.0±0.0


Analysis was performed using the Kruskal-Wallis test.

### Serum estradiol measurement

**Fig.2:**
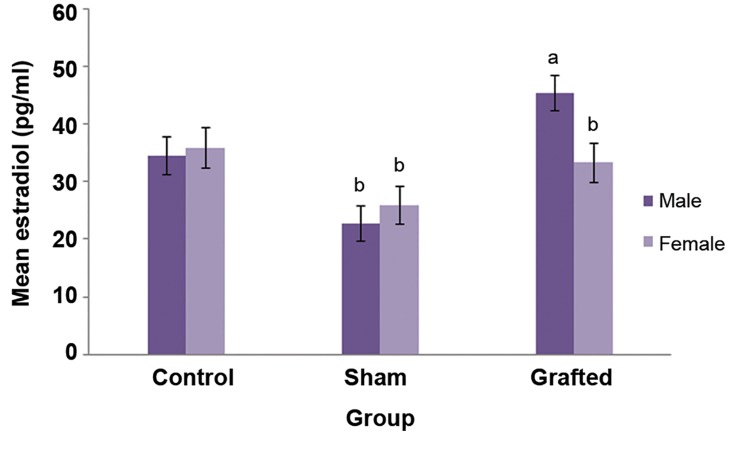
Estradiol (E_2_) secretion rate in the control and sham groups. Control group included 7 male and 7 female non-castrated/ non-grafted rats. Sham group included 7 male and 7 female castrated/non-grafted rats. In our xenograft model, there were 7 males and 7 females. Analysis was carried out using one-way ANOVA with a random effect. E_2_ level with different superscripts is significantly different (a, b) (P<0.05).

We observed a significant difference in E_2_ levels( pg/ml ) between the XM ( 45.44% ) and XF ( 33.36% ) groups. The XM group ( 45.44% ) compared to the sham male ( 22.71% ) and female ( 25.96% ) rats significantly differed ( P˂0.05, Fig.2 ). 

## Discussion

In this study, we have evaluated the survival and growth of sheep primordial follicles after xenotransplantation of sheep ovarian tissue into immunodeficient rats. Xenotransplantation of ovarian tissue is a promising procedure to preserve endangered animal species and a useful investigative tool to understand follicular development and ovarian physiology (12,18). Several breeds of animals with suppressed immune systems can act as recipients for xenotransplantation (19,21). 

In this study, we used rats as the host and incubator for follicular development. Rats were immunosuppressed following administration of cyclosporine A. To ensure the absence of rejection before xenotransplantation, each rat underwent blood testing. A study by Aubard (22) reported that the immune system of the recipient could be weakened during xenotransplantation by turning off the recipient’s blood immune response. With the immune system of the rats weakened with cyclosporine A, the rats were susceptible to infection and various diseases which resulted in the deaths of a number of rats. The percentage of follicles that survived after transplantation decreased in both male and female rats compared to the control group. The percentage of deaths and illnesses were higher in female rats compared to males. Ischemia-perfusion injury caused during the revascularization process was assumed to be the principal factor responsible for a severe reduction in the primordial follicle population in transplanted ovarian tissue (2,9,12,14,23). 

In an earlier study, primary follicles were expected to be found after xenotransplantation of human ovarian tissue to immunodeficient mice; however, antral follicles were observed just after injection of exogenous FSH (23). In fresh primate ovarian tissue xenotransplantation, FSH could improve the follicular number and their morphology by preserving the resting follicle pool. In another study administration of long-term FSH decreased the number of primordial follicles in xenografts of cryopreserved human ovarian tissue (24,25). Therefore, in the present study, we used hMG, not only as an exogenous gonadotropin, but also as a factor in angiogenesis to promote follicular development in grafted ovarian tissues according to Maltaris et al. (14). We have shown that exogenous gonadotropin increases the number of primary and preantral follicles rather than the resting follicles. A number of studies reported positive effects of mouse gonad removal and exogenous gonadotropin injections on the development of follicles in the ovary (2,12,14). Induction of revascularization and decrease in ischemia-perfusion are the best routes to maximize follicular survival. Recent studies (26,27) have shown a direct effect of gonadotropins on induction of endothelial cells for revascularization. Gonadotropins, including human chorionic gonadotropin ( hCG ), LH and FSH are factors for tissue-specific angiogenesis and hormones that regulate the vascular system (26). 

Other studies observed that male mice, with high concentrations of androgen, were better hosts for the development of growing follicles than female mice (28,29). They assumed that production of endogenous androgens could act as a substrate for estrogen, which could protect the antral follicles (2,12,13,28,29). 

However, this supposition did not apply in our study because the gonads were removed along with transplantation in both male and in female immunodeficient rats. In addition, hMG hormone injection in castrated male rats affected the hypothalamus and caused the secretion of gonadotropin-releasing hormone ( GnRH ). This hormone subsequently stimulates the pituitary gland and causes a normal rate LH and FSH secretion, whereas increasing hormone dosage in castrated female rats may stop or slow down the hormonal cycle. The percentage of follicles between transplanted groups and the control group showed no significant difference. The percent mean E ^2secre-^tion in grafted males was significantly higher than grafted females ( P˂0.05 ). 

## Conclusion

Despite significant differences in E_2_ value (pg/ ml) between xenografted male and female rats, we observed no statistical difference in terms of follicular development. We found no justification for follicular development and increasing E_2_ levels.

In general, castrated male rats, due to increased levels of E_2_ and a better survival rate, are likely to be better candidates for supporting ovarian xenotransplantations compared to castrated female rats.
